# Improving the Reliability of Protein Folding Rate Predictions by Applying Guidelines for Validating QSAR/QSPR Models

**DOI:** 10.3390/ijms27072968

**Published:** 2026-03-25

**Authors:** Antonija Kraljević, Jadranko Batista, Viktor Bojović, Bono Lučić

**Affiliations:** 1 Faculty of Mechanical Engineering, Computing and Electrical Engineering, University of Mostar, 88000 Mostar, Bosnia and Herzegovina; 2 Faculty of Science, Doctoral Study of Biophysics, University of Split, 21000 Split, Croatia; 3 Faculty of Science and Education, University of Mostar, 88000 Mostar, Bosnia and Herzegovina; 4 Ruđer Bošković Institute, 10000 Zagreb, Croatia

**Keywords:** protein folding rates, model validation, OECD guidelines, comparative analysis, web server, QSAR, QSPR, external validation, experimental data deviation, sequence length

## Abstract

Quantitative structure–activity/property relationship (QSAR/QSPR) is a well-established methodology widely used to model molecular properties based on structure and is applied in fields such as drug design and environmental protection. The knowledge and procedures developed and used in QSPR modelling will be applied to the validation of protein folding rate models. Understanding the protein folding process is considered one of the most important scientific topics, and identifying the fundamental factors responsible for protein folding has been the subject of intensive research over the past 30 years. Among the structural descriptors determining the protein folding rate, the length of the protein sequence, the content of regular secondary structures, and the average contact row distance between amino acids in the 3D structure are the most important. Comparative studies of different methods for predicting protein folding rates are occasionally published, and we conducted one such study. We found that the experimental data in literature databases and the data available online are inconsistent and scattered. This is partly due to differences in experimental data and protein sequence lengths, but more so due to the questionable quality of the models themselves. We observed very large deviations in the predictions of ln(*k_f_*) by some of the analysed models implemented as web servers. The root mean square errors (RMSEs) of some of the analysed models in predicting ln(*k_f_*) for a new external set of proteins are much larger than the RMSEs obtained for the same models on the training sets. External validation demonstrates that protein folding rate models available on web servers have accuracy for external protein sets comparable to that of a simple model based solely on the logarithm of protein chain length. This finding, which highlights the importance of external model validation as recommended by the OECD guidelines for QSAR validation, is fundamental and offers a new perspective for improving protein folding rate models by applying the knowledge and procedures used in the QSPR methodology.

## 1. Introduction

Since Anfinsen’s experiments, the problem of protein self-organisation has attracted the attention of physicists and has become a central issue in protein physics [1]. Although the mechanisms underlying this process remain unclear, researchers have devoted considerable time to studying folding kinetics to understand the relationship between the amino acid sequence and the three-dimensional (3D) structure of proteins [2], as the 3D, or native, structure is closely linked to protein function [3,4]. Only correctly folded proteins can become biologically active and perform their functions properly [5], whereas incorrectly folded proteins are implicated in many neurodegenerative diseases, such as Alzheimer’s disease and prion diseases [6].

The protein folding rate is a fundamental parameter describing the kinetics of protein folding. It is defined as *k_f_* = 1/*t*, where t is the time required for the protein to fold, and is typically represented on a logarithmic scale as ln(*k_f_*). Determining folding rates experimentally is challenging and has been achieved for only a limited number of proteins, so the number of proteins with experimentally determined folding rates is increasing slowly. One of the most commonly used protein sets in folding rate modelling and web server development included 80 proteins [7], while the recently published server FRTpred [8] was developed with a set of 141 proteins (111 for training and 30 for validation). This difficulty, together with the slow increase in the number of proteins for which the folding rate has been experimentally determined, has led to the growing importance of developing computer models based on available data and using them to estimate protein folding rates [1,9].

Predictors of protein folding rates use as features (descriptors) either information based on the protein sequence and its length, or information derived from the 3D structure of proteins (contact order, total contact distance, fraction of local contacts, chain topology parameter, geometric contact number, etc.) [1,7,9]. Prediction of protein folding rates by bioinformatics tools using information from the 3D structure of proteins has shown good correlation with experimentally measured protein folding rates [7,9]. However, such approaches are only suitable for broader applications to a limited extent, as prediction requires the experimentally determined or predicted 3D structure of the protein. In addition, due to the sensitivity of these methods to relatively frequent deviations in the file format containing the 3D structure, they sometimes fail to obtain ln(*k_f_*) predictions for certain proteins.

It is much more challenging to develop a model based on the known amino acid sequence, the structural class of a protein (determined by secondary structure estimation or prediction), the length of the protein sequence or the effective folding length [1], and other protein properties calculated from the amino acid sequence and the physicochemical properties of the 20 amino acids [10,11,12]. Prediction based solely on amino acid sequence can be readily applied to many natural, synthetic, and semi-synthetic protein sequences [9]. Some of these predictive models are available as online web servers, and we have tested their functionality and reliability in generating predictions. One such method, FRTpred [8], was recently published. It is based on the Random Forest (RF) algorithm and uses as inputs structural descriptors derived from the protein primary structure and predicted secondary structure to provide relatively accurate predictions of protein folding rates. Although the model structure in the FRTpred method is relatively complex and not easily interpreted, its advantage is that predictions can be obtained via a web server created and provided by the authors [8].

However, it is very common for a method to become unavailable just a few years after it is published and uploaded to a web server, often due to server malfunction. For example, of the eight web servers available online in 2014 [9], four are no longer in operation, possibly because of the cost of maintaining their functionality, changes in web server technology, security concerns, or changes in the author’s employment, when, as a rule, scientific supervision of the developed method ends. Therefore, in addition to more complex methods hosted on a web server and accessible via the Internet, it would be desirable to develop simpler models that can be used for predictions based on the model equations provided in the publication. One aim of this study is to develop simple models based solely on the length of the protein chain.

Of the eight methods analysed in the comparative study by Chang et al. [9], the training sets used in their development contained between 54 and 80 proteins. The expansion of training sets for model development is relatively slow; thus, the training set in the FRTpred method published in 2022 was larger, containing 111 proteins, while 30 proteins were used for external validation [8]. All the protein sets used for developing models to predict protein folding rates are relatively small and do not contain enough examples to train, optimise, and validate stable, complex models. Seven of the eight methods analysed in 2014 were mainly validated using the Leave-One-Out (LOO) Cross-Validation (CV) procedure, as indicated in Table 1 in [9]. The Pearson correlation coefficients (PCCs) obtained between estimated and experimental log_10_(*k_f_*) values of the training sets ranged from 0.74 to 0.93, while the RMSEs (Root Mean Square Errors) and Mean Absolute Errors (MAEs) reported for some methods ranged from 1.34 to 2.03 and from 0.23 to 1.19, respectively. It was stated that only one method (SeqRate [13]) was validated on an external test set of only seven proteins (Table 1 in [9]). However, upon reviewing the original publication, it was found that the final prediction of the SeqRate method is the average of the predictions of five models, each trained on a randomly selected 90% of the total number of proteins (and optimised in the LOO CV procedure), with its accuracy checked on the remaining 10% of proteins [13]. Therefore, the SeqRate method was also validated by a cross-validation procedure (leave k proteins out), but not on an external set. It remains unclear which proteins were involved in the validation of the final model and in the calculation of PCC, RMSE, and MAE. Additionally, it is evident that some proteins may have been selected more than once in some of the five test subsets, while some may not have been selected at all in some of the five test subsets.

In addition, the SeqRate method was applied to a set of 24 proteins that fold without intermediate states via two-state kinetics (denoted TS, i.e., two-state folder) and a set of 37 proteins that fold with one or more intermediate states via multi-state kinetics (denoted MS, i.e., multi-state folder), with separate models developed for each set. The SeqRate method [13] achieved the lowest MAE value in a comparative study of eight methods (MAE values of 0.79 and 0.68 on the log_10_ scale for TS and MS folders, respectively) [9].

For some methods, two sets of predictions can be obtained using either (1) a general model or (2) specific models. Specific models are developed for protein groups with the same protein kinetics (e.g., TS or MS [8,13]) or for proteins belonging to the same structural class [10]. Based on the amino acid residue content in regular secondary structures α or β, three structural classes of proteins are defined: all-α (α content >40% and β content <5%), all-β (β content >40% and α content <5%), and mixed class (protein contains at least 15% α-helices and at least 10% β-strands) [10]. The general model is developed regardless of factors such as structural class (i.e., proportion of regular secondary structures α or β) or type of folding kinetics (TS or MS) that may influence protein folding rates. For specific models, the initial set of proteins used in modelling is divided into two or more subsets. Each specific model is then developed (trained and validated) separately using only examples from the corresponding subset. However, splitting the relatively small initial set of proteins into even smaller subsets reduces the significance and stability of such models, as the model coefficients are derived from fewer examples. Another problem is that the secondary structure elements of a protein whose 3D structure has not yet been solved must be predicted using available bioinformatics methods, which will always involve some degree of class prediction error. The same applies to information about the kinetics of protein folding (TS or MS), which is always predicted with some error [8]. These two problems are worth mentioning because they are rarely addressed in the literature on protein folding rate modelling [8,9,10].

The above limitations of methods for predicting protein folding rates indicate that existing approaches require re-evaluation. Furthermore, previously identified dependencies of protein folding rates on structural parameters derived from protein sequence, physicochemical properties of amino acids (i.e., amino acid residues in proteins), 3D protein structure, and other derived parameters—such as those based on protein secondary structure or structural class—should be re-analysed. This study will implement more rigorous model validation procedures on external sets, as is standardly recommended in the field of QSAR (Quantitative Structure–Activity Relationship) and QSPR (Quantitative Structure–Property Relationship) for regulatory modelling of the activities and related properties of chemicals entering the environment [14,15]. The OECD (Organisation for Economic Co-operation and Development) principles for QSAR model validation were established after extensive discussions during the 1990s and 2000s within the QSAR community among leading experts in molecular modelling. This document was first defined in 2004 and updated in 2007 [15]. The OECD principles on QSAR model validation influenced subsequent work by QSAR modellers and led to significant improvements in the validation of QSAR molecular models used in regulatory fields related to environmental and human health protection, as well as other areas. This document briefly describes the history and development of applying models to predict toxicity and related endpoints (such as acute toxicity, carcinogenicity, and mutagenicity) from the structure of chemical compounds while reducing the need for experiments on living organisms such as vertebrates or mammals (Non-Animal Testing Methods, NAMs).

The number of optimised parameters in the analysed methods will be estimated, and their stability in predicting protein folding rates will be compared. Furthermore, we developed the simplest models based solely on protein sequence length and assessed the impact of deviations in the input experimental data (folding rate and protein sequence length) on the performance of these models. Finally, we will compare and discuss all available methods used in this study, considering their complexity, accuracy, and stability of predictions. Some complex methods exhibit unexpectedly large uncertainties (errors) in prediction, while the simplest models developed in this study, based only on protein sequence length, provide stable predictions for independent test sets of proteins, with errors comparable to those on the training sets.

## 2. Results and Discussion

### 2.1. General Overview of the Comparison of Various Methods in Prediction of ln(k_f_) of Proteins

To independently and externally verify the accuracy/quality of protein folding rate prediction methods, we created protein sets by determining the intersection and difference of two protein sets (as described in Section 3.2) from three sets (S77, S80, and S111), for which four methods have been developed and are available as functional web servers. In this way, we obtained three pairs of sets to compare the methods, which are summarised in Table 1, Table 2 and Table 3. The corresponding protein sets and subsets used at each step of the analysis and comparison are listed in Supplementary Tables S1–S3 for the pairs S77–S80, S77–S111, and S80–S111. These tables show the results of (1) the original methods available via four web servers for the subsets (models) described in Section 3.2 and (2) our own results (models) in the sections labelled ‘Our model’ in Table 1, Table 2 and Table 3. The Supplementary Tables S1–S3 contain experimental and predicted ln(*k_f_*) values of proteins, together with their names and sequences. They are organised into three tables in Microsoft Excel format, each with multiple layers, and can be used to reproduce results and for comparative analyses.

Our results presented in Table 1, Table 2 and Table 3 refer to models based solely on the length of the protein sequence, *L* or ln(*L*) (i.e., the number of amino acids in the protein chain), and have abbreviations beginning with ‘MO’ (‘Model-Our’). The abbreviations for results referring to models available via the web server begin with ‘MS’ (‘Model-Server’). Additionally, the names of the results obtained via the server are numbered from 1 to 4 (MS.1 to MS.4) for FOLD-RATE, Pred-PFR, FoldRate, and FRTpred, respectively. Our results have numerical designations 1 to 6 (MO.1 to MO.6), two for each of Table 1, Table 2 and Table 3, because there were certain differences in the experimental data for the group of overlapping proteins (present in both groups analysed)—that is, differences in the lengths or compositions of the sequences and differences in the ln(*k_f_*) values. To test the impact of these differences on the final models and results, we also performed modelling using the experimental data from the first and second sets from each observed pair of sets. For example, in Table 1, we developed the first model (MO.1) for the overlapping proteins in sets S77 and S80 (set S77 ∩ S80) using experimental data (for each protein) from set S77. We also developed the second model (MO.2) for the overlapping proteins using experimental data from set S80, and we labelled this overlap as set S80 ∩ S77.

### 2.2. Comparative Analysis of Protein-Folding Rate Models on Protein Sets S77 and S80, and Their Overlapping Subsets

Prediction results of the methods FOLD-RATE [10], Pred-PFR [12], and FoldRate [11], as well as details of models developed for combinations of the S77 and S80 datasets, are provided in Table 1. These datasets, taken from [10] and [7], contain 77 and 80 proteins, respectively.

**Table 1:** Statistical measures of folding rate models (evaluation metrics) from the literature [7,10,12], developed on the S77 (77 proteins) and S80 (80 proteins) datasets, and models from this study based on ln(*L*), developed on the overlapping sets of S77 and S80 ^a^.

Model No.	Method Acronym, Dataset ^b^	n ^c^	RMSE	PCC	RMSE_PR_	PCC_PR_	RMSE_cv_	PCC_cv_
MS.1	FOLD-RATE_S77, [10]	77	1.84	0.87			1.65	0.863
MS.1a	S77 ∩ S80	52	1.96	0.862				
MS.1b	S80 ∩ S77	50	2.87	0.740				
MS.1c	S80\S77	30			7.81	−0.306		
MS.2	Pred-PFR_S80 ^d^ [12]	80	2.06 (*n* = 72)	0.853 (*n* = 72)			2.03 (*n* = 80)	0.88 (*n* = 80)
MS.2a	S80 ∩ S77	47	2.08	0.820				
MS.2b	S77 ∩ S80	50	2.54	0.729				
MS.2c	S77\S80	24			3.31	0.466		
MS.3	FoldRate_S80 ^d^ [11]	80	2.06 (*n* = 72)	0.853 (*n* = 72)			2.03 (*n* = 80)	0.88 (*n* = 80)
MS.3a	S80 ∩ S77	47	2.04	0.826				
MS.3b	S77 ∩ S80	50	2.47	0.745				
MS.3c	S77\S80	24			3.53	0.393		
MO.1	Our-Model-1_S77 ^b,d^ ln(*k_f_*) = 30.788 − 5.985·ln(*L*) S77 ∩ S80	52 50	2.80	0.685			2.91	0.654
MO.1a	S77\S80	25			3.89	0.246		
MO.1b	S80\S77	30			2.89	0.819		
MO.2	Our-Model-2_S80 ^b,d^ ln(*k_f_*) = 32.439 − 6.411·ln(*L*) S80 ∩ S77	50	2.46	0.770			2.54	0.753
MO.2a	S80\S77	30			2.87	0.819		
MO.2b	S77\S80	25			4.05	0.246		

^a^ S77 and S80 are datasets from [10] and [7], containing 77 and 80 proteins, respectively. Overlapping sets share proteins with identical PDB codes. Due to differences in amino acid sequences and experimental folding rates for proteins with the same PDB codes, two options were considered for overlapping sets: (1) use data from S77 (designated as S77 ∩ S80) or (2) use data from S80 (designated as S80 ∩ S77). ^b^ FOLD-RATE_S77, Pred-PFR_S80 [12] and FoldRate_S80 [11] are models (methods) available via web servers. They were developed using experimental data from datasets S77, S80 and S80, respectively. Our-Model-1_S77 and Our-Model-2_S80 are models from this study developed using the overlapping set of experimental data from dataset S77 (for S77 ∩ S80) and S80 (for S80 ∩ S77), respectively. The Methods section provides a detailed explanation of acronyms and datasets. ^c^ *n* is the number of data points, i.e., the number of overlapping proteins between S77 and S80. The meanings of the acronyms for the statistical measures are explained in the Methods section. ^d^ *L* is the length of a protein sequence (the number of amino acids in a sequence). The models developed in this study have the general form ln(*k_f_*) = *a*_0_ + *a*_1_·ln(*L*).

Overlapping sets are constructed based on their PDB codes and share common proteins. As described in the Methods section, due to differences in amino acid sequences and experimental folding rates of proteins with identical PDB codes, there are two options for overlapping sets: (1) using data from S77 (labelled S77 ∩ S80) or (2) using data from S80 (labelled S80 ∩ S77). Dataset S77 contains two protein sequence pairs (1UBQ and 2A5E) that appear twice with identical sequences but different experimental folding rates. As a result, the subset S77 ∩ S80 consists of *n* = 52 proteins, while the subset S80 ∩ S77 contains *n* = 50 proteins.

Three methods available on the web servers listed in Table 1 (i.e., FOLD-RATE_S77 [10], Pred-PFR_S80 [12], and FoldRate_S80 [11]) were developed using experimental data from datasets S77, S80, and S80, respectively. These methods are denoted in Table 1 as models MS.1, MS.2, and MS.3. When reproducing the results for the latter two methods, there is a limitation in that the two servers only provide predictions for proteins with a length *L* ≥ 50 amino acid residues, which reduces the expected S80 dataset to *n* = 72 proteins. Consequently, for models MS.2a and MS.3a, the subset S80 ∩ S77 contains *n* = 47 instead of *n* = 50 proteins; for models MS.2b and MS.3b, the subset S77 ∩ S80 contains *n* = 50 instead of *n* = 52 proteins. Finally, for models MS.2c and MS.3c, the subset S77\S80 contains *n* = 24 instead of the expected 25 proteins.

Table 1 presents our linear models, labelled MO.1 and MO.2, of the general form ln(*k_f_*) = *a*_0_ + *a*_1_·ln(*L*), developed for the subsets S77 ∩ S80 (*n* = 52) and S80 ∩ S77 (*n* = 50), respectively. The quality of these models can be assessed using statistical measures (RMSE and PCC) calculated during the fitting procedure, LOO CV, and prediction on external protein datasets, with the latter (RMSE_PR_, PCC_PR_) considered the most important.

Model MS.1 in Table 1 was developed using the entire S77 set and is presented in the literature by Gromiha et al. [10]. This method, named FOLD-RATE, is implemented on the server and abbreviated as FOLD-RATE_S77. The RMSE (1.84) and PCC (0.87) values of the MS.1 model obtained during the fitting procedure match those reported in the cited publication, as do the corresponding LOO CV values (1.65 and 0.863, respectively). These fitted and LOO CV RMSE and PCC values differ only slightly, indicating model stability and suggesting it is neither overfitted nor burdened with too many optimised parameters.

The statistical measures RMSE (1.96) and PCC (0.862) for the MS.1a model, calculated for the overlapping subset S77 ∩ S80 of the S77 and S80 sets with 52 proteins (*n* = 52), using the experimental data (protein sequences and ln(*k_f_*) values) from the S77 set [10], are very close to the RMSE and PCC values obtained for the whole set. However, they differ significantly (MS.1b model on the set S80 ∩ S77) when experimental data from the S80 set are used (RMSE = 2.87, PCC = 0.74), which may indicate overfitting of the model to the initial training set S77 data. Furthermore, the most stringent test of the model’s predictive accuracy is the prediction results on the S80\S77 set of 30 proteins given in Table 1 (row MS.1c), which were not used in the development of the MS.1 model, with experimental data from the reference in which the S80 set was published [7]. The RMSE (7.81) and PCC (−0.306) values deviate significantly from the corresponding values given in Table 1 for models MS.1, MS.1a, and MS.1b. RMSE_PR_, which is four times higher than the RMSE on the training set, indicates overfitting of the model to the S77 protein set data on which it was developed, resulting in excessive deviation of ln(*k_f_*) predictions for new proteins from the external test set.

The Pred-PFR_S80 method (model MS.2) was developed for the S80 protein set and uploaded to the Internet server [12]. It is a consensus or ensemble method (Equation (8) in [12]) that provides a prediction as the average of seven individual linear models, each including one or more descriptors (structural parameters) and optimising two or more model parameters. All these individual models, which form the final ensemble model Pred-PFR_S80, are based on the physical properties of amino acids, such as affinity for alpha or beta secondary structures, compressibility, solvent-accessible surface area in an unfolded protein chain, protein depth and effective length, or the proportion of alpha, beta and irregular secondary structures in proteins. The RMSE value for the MS.2 model underlying this method on the server is 2.06 (Table 1), which is close to the corresponding values calculated on the subsets S80 ∩ S77 (model MS.2a: 2.08, *n* = 47) and S77 ∩ S80 (model MS.2b: 2.54, *n* = 50), as well as on the external protein set S77\S80 (model MS.2c: 3.31, *n* = 24).

Using the FoldRate_S80 server (Table 1), which is based on the S80 set [11], we obtained estimates and predictions of ln(*k_f_*) that are very similar to those obtained with the Pred-PFR_S80 method. Models MS.3, MS.3a, and MS.3b have RMSE and PCC values very similar to those of the corresponding models MS.2, MS.2a, and MS.2b described above and obtained by Pred-PFR_S80. Additionally, the prediction by the MS.3c model (RMSE = 3.53, *n* = 24) on the external test set S77\S80 is very similar to that obtained by the MS.2c model. Therefore, we conclude that the Pred-PFR_S80 and FoldRate_S80 methods are less fitted to proteins from their training set S80 and have better predictive properties than the FOLD-RATE_S77 method [10]. An unusual coincidence is that the values of RMSE_cv_ (2.03) and PCC_cv_ (0.88) are identical for the FoldRate_S80 and Pred-PFR_S80 methods (MS.2 and MS.3, Table 1), although these methods are based on different models that share some similarity. These two methods were developed on the same set of proteins, by the same research group, and published in the same year. According to the publisher’s data, the FoldRate_S80 method is somewhat older [11]. As mentioned above, the Pred-PFR_S80 method is an ensemble of seven individual models [12], while the FoldRate_S80 method is an ensemble of three individual linear models based on: (1) the length of the protein (i.e., ln(*L*)), (2) the logarithm of the effective length of the protein, and (3) the average propensity of amino acids in the protein to form a beta secondary structure [11]. The first two individual models in the FoldRate_S80 method are also included in the Pred-PFR_S80 method, while the third individual linear model is included in one of the seven individual models within the Pred-PFR_S80 method, along with the propensities of amino acids in the protein to form beta and coil secondary structures.

In parts/layers (A–H) of Table S1, detailed results of modelling ln(*k_f_*) on sets S77 (Table S1A) and S80 (Table S1B), as well as on subsets obtained from their overlaps (Table S1C–F), are provided. Each value presented in Table 1 can be reproduced from the data in the corresponding parts of Table S1.

### 2.3. Comparative Analysis of Protein-Folding Rate Models on Protein Sets S77 and S111, and Their Overlapping Subsets

Table 2 presents the results of methods from the literature used to model and predict ln(*k_f_*) for protein subsets derived from the S77 and S111 datasets using web servers FOLD-RATE [10] and FRTpred [8]. It also includes our models based solely on protein sequence length (i.e., ln(*L*)), developed on the overlapping subsets S77 ∩ S111 (*n* = 42, model MO.3) and S111 ∩ S77 (*n* = 40, model MO.4).

Model MS.1 in Table 2 is associated with the web server FOLD-RATE [10]. It is abbreviated as FOLD-RATE_S77, developed on the S77 set, and is also listed in Table 1 and described above. Here, model MS.1 is applied to the new subsets S77 ∩ S111 (MS.1d) and S111 ∩ S77 (MS.1e), obtained from the overlap of the S77 and S111 sets. If the experimental protein sequence data and protein folding rates for each pair of proteins (matched by their PDB code) differ, the data from the first listed set (S77 in the S77 ∩ S111 subset and S111 in the S111 ∩ S77 subset) are used. The set S77 ∩ S111 is a subset of S77 on which the FOLD-RATE_S77 method was developed. The errors of the MS.1 model on these two subsets are 1.65 for MS.1d and 2.42 for MS.1e, both slightly lower than the RMSE values of the corresponding models MS.1a (1.96) and MS.1b (2.87) in Table 1. The differences in the experimental data cause a significant increase in the error, indicating the need for more precise data, but also suggesting possible overfitting of the FOLD-RATE method to the S77 dataset on which it was developed.

Most importantly, the RMSE_PR_ in the prediction of ln(*k_f_*) values for 71 new proteins from the set S111\S77 is 6.31 (MS.1f), which is comparably high to the RMSE_PR_ value of the MS.1c model from Table 1, obtained on the subset S80\S77, which is 7.81. Furthermore, this error is two to three times higher than the RMSE values for MS.1d and MS.1e obtained on the subsets S77 ∩ S111 and S111 ∩ S77, indicating overfitting of the FOLD-RATE method to the data on which it was developed.

The second model, MS.4 in Table 2, is based on the FRTpred_S111 web server, which was developed from the S111 protein set [8]. This method is a meta-predictor that uses the Random Forest (RF) algorithm, named mRF (metaRF), and is optimised on 30 baseline models. These baseline models were constructed using 10 different feature encodings and three Machine Learning (ML) methods: Extremely Randomised Tree (ERT), Support Vector Machine (SVM), and Random Forest (RF). The FRTpred method is evidently the most robust of all the methods analysed in this study, is highly non-linear, and optimises a large number of parameters. Interpreting the model in physico-chemical terms is very difficult and, if possible, it would be extremely challenging to trace the interdependence of the input features given their multitude. Optimisation of the model underlying the FRTpred method was performed using the LOO CV procedure on the S111 training set. An independent set of 30 proteins (S30) was used to assess the quality of the model and to compare it with three methods for predicting ln(*k_f_*) that were available as web servers at that time. This comparison showed higher accuracy of the FRTpred method compared to competing methods (Table 3 in ref. [8]).

**Table 2:** Statistical measures of folding rate models from the literature [8,10] developed on datasets S77 (77 proteins) and S111 (111 proteins), and models from this study based on ln(*L*) developed on the overlapping sets of S77 and S111 ^a^.

Model No.	Model Acronym_Data Set ^b^	n ^c^	RMSE	PCC	RMSE_PR_	PCC_PR_	RMSE_cv_	PCC_cv_
MS.1	FOLD-RATE_S77 ^a^, [10]	77	1.84	0.871			1.65	0.863
MS.1d	S77 ∩ S111	42	1.65	0.891				
MS.1e	S111 ∩ S77	40	2.42	0.807				
MS.1f	S111\S77	71			6.31	0.050		
MS.4	FRTpred_S111 ^a^, [8]	111	1.47	0.936			1.954	-
MS.4a	S77 ∩ S111	42	1.91	0.864				
MS.4b	S111 ∩ S77	40	1.50	0.923				
MS.4c	S77\S111	35			2.59	0.750		
MO.3	Our-Model-3_S77 ^b,d^ ln(*k_f_*) = 24.140 − 4.496·ln(*L*) S77 ∩ S111	42	2.99	0.538			3.09	0.525
MO.3a	S77\S111	35			3.30	0.556		
MO.3b	S111\S77	71			3.22	0.757		
MO.4	Our-Model-4_S111 ^b,d^ ln(*k_f_*) = 24.621 − 4.587·ln(*L*) S111 ∩ S77	40	2.97	0.575			3.16	0.464
MO.4a	S111\S77	71			3.19	0.757		
MO.4b	S77\S111	35			3.29	0.556		

^a^ S77 and S111 are data sets from [10] and [8] containing 77 and 111 proteins, respectively. For more details, see footnote ‘a’ of Table 1. ^b^ FOLD-RATE_S77 and FRTpred_S111 [8] are models/methods that are available via web servers. Our-Model-3_S77 and Our-Model-4_S111 are models from this study developed using the overlapping set of experimental data from dataset S77 (for S77 ∩ S111) and S111 (designated as S111 ∩ S77), respectively. For more details see Methods and footnote ‘b’ of Table 1. ^c^ *n* is the number of data points (for more details, see footnote ‘c’ of Table 1). ^d^ *L* is the length of a protein sequence (for more details, see footnote ‘d’ of Table 1).

The errors of model MS.4 on S111 (RMSE = 1.47) and on the subset S111 ∩ S77 (MS.4b, RMSE = 1.50) in Table 2 correspond to the fitting errors for the proteins from the training set, and are somewhat lower than those reported in the original publication (RMSE = 1.954, as given in the abstract of [8]), as the latter were obtained using a slightly stricter LOO CV validation procedure. Using the experimental values from the S77 set to evaluate accuracy on the S77 ∩ S111 subset yields a slightly higher error (MS.4a, RMSE = 1.91), indicating a significant influence of experimental data variation on the reported model accuracy. Finally, the prediction by the FRTpred_S111 method on 35 proteins from the non-overlapping subset S77\S111 yields a noticeably higher error value (MS.4c, RMSE = 2.59), which should be compared with the values on the training set calculated during the fitting procedure (1.47) and LOO CV (1.954).

Simple linear models based solely on protein length were developed for the overlapping subsets S77 ∩ S111 (*n* = 42, MO.3, RMSE = 2.99) and S111 ∩ S77 (*n* = 40, MO.4, RMSE = 2.97). Predictions on independent sets of 35 proteins (MO.3a, RMSE = 3.30; MO.4b, RMSE = 3.29) and 71 proteins (MO.3b, RMSE = 3.22; MO.4a, RMSE = 3.19) for the MO.3 and MO.4 models showed errors only slightly higher than those obtained during fitting on the training sets. The RMSE values for the MO.3 (3.09) and MO.4 (3.16) models calculated using the LOO CV procedure were also only slightly higher than the corresponding fitting values (2.99 and 2.97). The similar values of model errors in fitting (RMSE), LOO CV (RMSE_CV_), and prediction (RMSE_PR_) on independent sets indicate the stability of simple linear models based on the logarithm of protein length (ln(*L*)).

A comparison of prediction errors on independent sets (RMSE_PR_) for all methods from Table 2 shows that the lowest error was achieved by the FRTpred method (2.59), followed by the models developed in this work based only on ln(*L*) (in the range 3.19–3.30), while the least reliable models in prediction were the MS.1 model and the FOLD-RATE method (6.31).

In parts/sheets (A–H) of Table S2, detailed results of modelling ln(*k_f_*) on sets S77 (Table S2A) and S111 (Table S2B), as well as on subsets obtained from their overlaps (Table S2C–F), are provided. Each value presented in Table 2 can be reproduced from the data in the corresponding parts of Table S2.

### 2.4. Comparative Analysis of Protein-Folding Rate Models on Protein Sets S80 and S111, and Their Overlapping Subsets

In the third part of the comparison of methods for modelling and predicting ln(*k_f_*), we analyse the results obtained for the S80 and S111 sets, as well as their overlapping and non-overlapping subsets. Overall, the error values and correlation coefficients in Table 3 follow the patterns observed in Table 1 and Table 2.

The Pred-PFR_S80 and FoldRate_S80 methods are similar and yield error values in the fitting procedure on the training sets in the range 1.35–2.15 (MS.2d, MS.2e, MS.3d, MS.3e, Table 3). In the LOO CV procedure, both methods give the same error (2.03). In prediction on the independent S111\S80 set of 58 proteins, the Pred-PFR method (MS.2f) achieves an RMSE of 3.21, while the FoldRate method (MS.3f) gives 3.35. The prediction errors are significantly higher, indicating some instability in these methods.

The FRTpred method (MS.4, MS.4d, MS.4e, Table 3), being more robust than all other methods analysed in this study, gives errors in the range 1.47–1.69 on the training set and subsets in the fitting procedure, and 1.954 in the LOO CV procedure. On the independent set S80\S111 of 29 proteins, the error of model MS.4 is 2.02 (results MS.4f), which is the lowest RMSE_PR_ value in Table 3.

The errors of the MO.5 and MO.6 models, based only on ln(*L*), in prediction on the independent subsets with 29 proteins (S80\S111, MO.5a, MS.6b) and 60 proteins (S111\S80, MO.6a, MS.5b), are in the narrow range of 2.54–2.86, and are comparable to the values obtained in the fitting (2.71, 2.72) and the LOO CV procedure (2.81), indicating the stability of these simple models in predicting new proteins.

The results in Table 3 confirm what was observed in Table 1 and Table 2: the available methods for predicting protein folding rates are less stable in predicting ln(*k_f_*) for new proteins than the simple models based solely on ln(*L*) developed and presented in this study.

**Table 3:** Statistical measures of folding rate models (evaluation metrics) from the literature [7,8,11,12] developed on the S80 (80 proteins) and S111 (111 proteins) datasets, and models from this study based on ln(*L*) developed on the overlapping sets of S80 and S111 ^a^.

Model No.	Model Acronym_Data Set ^b^	n ^c^	RMSE	PCC	RMSE_PR_	PCC_PR_	RMSE_cv_	PCC_cv_
MS.2	Pred-PFR_S80 ^d^ [12]	80	2.06 (*n* = 72)	0.853 (*n* = 72)			2.03 (*n* = 80)	0.88 (*n* = 80)
MS.2d	S80 ∩ S111	46	1.35	0.852				
MS.2e	S111 ∩ S80	45	2.15	0.823				
MS.2f	S111\S80	58			3.21	0.727		
MS.3	FoldRate_S80 ^d^ [11]	80	2.06 (*n* = 72)	0.853 (*n* = 72)			2.03 (*n* = 80)	0.88 (*n* = 80)
MS.3d	S80 ∩ S111	46	2.12	0.853				
MS.3e	S111 ∩ S80	45	2.08	0.833				
MS.3f	S111\S80	58			3.35	0.711		
MS.4	FRTpred_S111, [8]	111	1.47	0.936			1.954	-
MS.4d	S111 ∩ S80	51	1.60	0.938				
MS.4e	S80 ∩ S111	51	1.68	0.938				
MS.4f	S80\S111	29			2.02	0.913		
MO.5	Our-Model-5_S80 ^b,e^ ln(*k_f_*) = 35.521 − 7.068·ln(*L*) S80 ∩ S111	51	2.71	0.767			2.81	0.748
MO.5a	S80\S111	29			2.54	0.848		
MO.5b	S111\S80	60			2.86	0.646		
MO.6	Our-Model-6_S111 ^b,e^ ln(*k_f_*) = 35.888 − 7.111·ln(*L*) S111 ∩ S80	51	2.72	0.766			2.81	0.746
MO.6a	S111\S80	60			2.80	0.646		
MO.6b	S80\S111	29			2.59	0.848		

^a^ S80 and S111 are data sets from [11] and [7], containing 80 and 111 proteins, respectively. For more details, see Methods and footnote ‘a’ of Table 1. ^b^ FoldRate_S80 and Pred-PFR_S80 [12] are models or methods developed using experimental data from dataset S80, and FRTpred_S111 is a model or method developed using experimental data from dataset S111. These methods are available via web servers. Our-Model-5_S80 and Our-Model-6_S111 are models from this study, developed using the overlapping set of experimental data from dataset S80 (for S80 ∩ S111) and S111 (designated as S111 ∩ S80), respectively. ^c^ *n* is the number of data points, i.e., the number of overlapping proteins in S80 and S111 (for more details, see footnote ‘c’ of Table 1). ^d^ Models FoldRate_S80 and Pred-PFR_S80 were developed on S80, but both servers can provide predictions only for 72 proteins with length *L* ≥ 50 residues. ^e^ *L* is the length of a protein sequence (the number of amino acids in a sequence). For more details, see footnote ‘d’ of Table 1.

In parts/layers (A–H) of Table S3, detailed results of modelling ln(*k_f_*) on datasets S80 (Table S3A) and S111 (Table S3B), as well as on subsets obtained from their overlaps S80 ∩ S111, S111 ∩ S80, S80\S111, and S111\S80 (Table S3C–F, respectively), are provided. Each RMSE and PCC value, along with our models presented in Table 3, can be calculated and reproduced from the data in the corresponding parts of Table S3.

### 2.5. Comparison of Protein Folding Rate Models Available Through Web Servers

#### 2.5.1. Comparative Analysis of Web Servers and Our Models Based on ln(*L*) in Modelling ln(*k_f_*)

To evaluate the quality of predictions for protein folding rates ln(*k_f_*), we used the S30 set of 30 proteins from the server and supplementary information of the article by Manavalan and Lee [8], which was also used as a prediction set by the authors of the FRTpred method. According to the type of folding process, the S30 set contains 12 non-two-state and 18 two-state folder proteins. PDB codes, protein sequences, and ln(*k_f_*) values for the S30 set are provided in Table S4. In Table S4A, the predicted ln(*k_f_*) values for S30 obtained using FOLD-RATE, FoldRate, Pred-PFR, and FRTpred (combined version) are shown.

Some of these methods could not predict protein sequences with fewer than 50 amino acid residues, so the results are presented without these proteins, i.e., for set S29. Table 4 shows the RMSE_PR_ and PCC_PR_ values for the four methods used: MS.1g, MS.2g, MS.3g, and MS.4g. The lowest RMSE_PR_ of 2.22 (model MS.4g) is achieved by FRTpred, the most robust method, developed as an ensemble of several single models. In addition, FRTpred is the only method in this study that used set S30 to test the predictive performance of all developed models.

The remaining models in Table 4, marked as MO.1, MO.2, …, MO.6c, were developed in this study and are based only on the logarithm of the length of the protein sequence, ln(*L*). The RMSE_PR_ values of models available through web servers for set S30 range from 2.22 to 5.77, while simple linear models based on ln(*L*) for prediction on new sets of proteins range from 2.83 to 3.34. We conclude that the analysed models are comparable in their predictions on new proteins. However, models based on ln(*L*) have a simple linear form with only two optimised parameters and are much easier to interpret physico-chemically.

Among the models underlying the analysed servers, as well as other models in the literature, the most significant correlations are achieved with parameters such as sequence length, average values of physicochemical properties of amino acids in the protein sequence [10,11,12], and especially properties calculated based on 3D structure, such as contact order distance. However, in most complex parameters, the contribution of protein length is present to a greater or lesser extent in both linear and nonlinear forms of the model. Therefore, if length and other variables (descriptors) correlated with length are used together in a more complex model, the problem of excessive intercorrelation of input variables arises, which introduces instabilities into the model. Such instabilities are most pronounced when the model is applied to external sets of proteins. The most commonly used set in the literature, S80, was introduced in a study by Ouyang and Liang [7], in which the best model for predicting protein folding rates is based on the geometric contact number, defined as the number of nonlocal contacts in the 3D protein structure that are well packed according to a Voronoi criterion. Analysis of the correlation between such a parameter and protein length in the S80 set shows a significant correlation (PCC = 0.91, n = 80). Therefore, to increase the stability of more complex models, it is necessary to introduce an appropriate restriction on the intercorrelation between the input variables.

The noticeably lower prediction quality on external sets compared to the estimate of ln(*k_f_*) for most of the more complex models analysed in this study could be prevented or mitigated either by reducing model complexity or by expanding the set of proteins used for training. The problem is that expanding the protein set requires additional measurements of protein folding rates on new protein sequences whose primary structures are not too similar to those in existing data sets. However, this is difficult to achieve, as the number of new proteins for which both the mechanism and folding rates have been measured increases very slowly and has not changed over the past 30 years.

#### 2.5.2. Comparison with RMSE and PCC Values of Published Models

There are minor differences between the statistical measures of model quality (RMSE, MAE, or PCC) obtained using web servers and the corresponding values reported in the literature, that is, in the original publications (see Table 5).

These differences are partly because neither FoldRate nor Pred-PFR can predict the folding rates of proteins with fewer than 50 amino acids. For the FOLD-RATE server [10], there are no restrictions on protein length, but the predicted folding rates vary greatly depending on the choice of structural class, which may be all-α, all-β, mixed, or unknown. A very high PCC value of 0.99 is observed for the FOLD-RATE method. Considering the overall analysis of the results in Table 1, Table 2, Table 3 and Table 4, this value is likely unrealistically high and may indicate overfitting of the model to the training data.

However, we observed that sometimes inappropriate names for statistical measures of model quality are used [9]. The authors of that comparative study made an oversight: Table 1 presents the MAE values (correctly calculated but incorrectly labelled as MAD, i.e., Mean Absolute Deviation) for all methods on a log_10_ scale, while Table 2 shows the MAE values on the natural logarithm scale (i.e., ln *x* = log_e_ *x*) calculated from the experimental and predicted folding rates on an independent test set. The authors provided the predicted values for each method in the supplementary information table of the comparative study [9]. From these values, we were able to calculate PCC, RMSE, or MAE. In the comparative study of eight methods available via the web server, the authors incorrectly labelled MAE as MAD (Mean Absolute Deviation) [9], while the authors of the SeqRate method [13] also used the abbreviation MAD but for the parameter Mean Absolute Difference (which measures the absolute deviation of the predicted values from the actual values [9]), which corresponds to MAE. By repeating the calculations based on the data in the supplementary materials of ref. [9], we found that the MADs in Table 2 in ref. [9] are identical to the MAEs. The best method on an independent test set of 28 proteins was SeqRate [13] (MAE = 2.7), followed by K-Fold [16] (MAE = 4.5, Table 2 in [9]).

**Figure 1:**
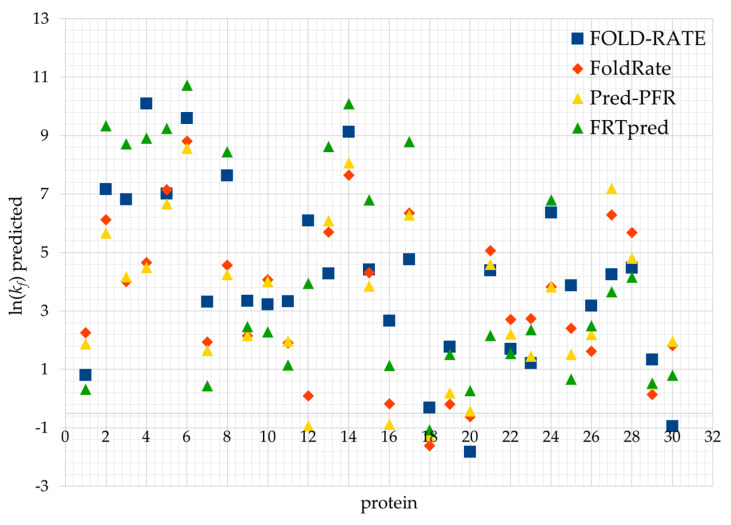
Predicted values of the folding rates for the four individual prediction tools for the overlapping set of 30 proteins.

In addition, we created an overlapping set of proteins from sets S77, S80 and S111 for all four prediction tools FOLD-RATE, Pred-PFR, FoldRate and FRTpred. Figure 1 shows the predicted folding rate values, ln(*k_f_*), for this set of 30 proteins. For some proteins, there is greater variation in the predicted values of ln(*k_f_*), indicating the need for further improvement of the model for predicting protein folding rates.

### 2.6. Physical Interpretation of Protein Folding Rate Models

In modelling, including molecular modelling, it is desirable to develop reliable and accurate models that can generalise to predict new examples, such as molecules or macromolecules. Ideally, each model should also be simple and interpretable, enabling understanding of the relationship between the predicted value (output) and the input variables describing the problem. For models that describe the relationship between a molecule’s structure, represented by structural descriptors (attributes), and one or more selected properties of the molecule, it is particularly important to be able to interpret the model in a physicochemical sense. Clear interpretation based on theoretical foundations and fundamental concepts allows better understanding of the problem and facilitates the design of molecules with improved or desired properties. Interpretability is especially important for models addressing key scientific problems, such as protein folding and the modelling of protein folding rates, which have been studied for many years. In this context, the models analysed and developed in this study can be categorised as: (1) complex multivariate linear and nonlinear models based on multiple input parameters and (2) simple linear models based on the length of the protein sequence.

The complex models FOLD-RATE [10], Pred-PFR [12], FoldRate [11], and FRTpred [8] are difficult to interpret because they are multivariate and the variables used are mutually correlated (i.e., not orthogonal). The first three methods are linear multivariate, while the last method (FRTpred) is nonlinear and multivariate. For the FOLD-RATE method [10], neither the publication nor the web server specifies the necessary details about the number and type of descriptors in the models by individual classes, or in the global model developed on the entire S77 training set. It is only stated that 10 descriptors are included in the global model developed on the S77 set, which is available on the FOLD-RATE server [10]. For the remaining three models, it can only be roughly assumed that the type and number of structural protein descriptors are similar to those in the authors’ previous publications [17,18]. Details of the models available on the Pred-PFR, FoldRate, and FRTpred servers are described in Section 3.1. They share the characteristic of being ensembles of several models, and each of which includes several descriptors (input variables) in linear (Pred-PFR and FoldRate) or highly nonlinear form (FRTpred). An additional problem is that in these models, it is not possible to clearly separate and evaluate the influence of protein sequence length, which has long been known from theory to be a very important determinant of protein folding rate [2,19], and the actual contributions of sequence length are often evaluated as late as possible in numerous studies [1,20,21]. Groups such as D. Baker [21,22] and M. Karplus [23,24] have also addressed the problem of protein folding rate, mainly investigating the influence of established contacts in the 3D structure of proteins between non-adjacent (distant) amino acids in the sequence. The specificity of these models is that they require a 3D protein structure, which reduces generality and limits their application. More complex nonlinear models have also been developed using neural network algorithms, starting from input structural protein descriptors, but with relatively small sets of 33 [24] and 28 proteins [25].

On the other hand, simple models based on ln(*L*), such as those developed in this study in the form presented in Equation (8) (Section 3.2), are much more easily interpreted. To clarify this further, we transform the model from Equation (8):

ln(*k_f_*) = ln(*c*_0_) + ln[(*L*)^*a*_1_^] (1)

where ln(*c*_0_) = *a*_0_. Further transformation gives the form shown in Equation (2):

ln(*k_f_*) = ln[*c*_0_·(*L*)^*a*_1_^], (2)

Finally, we obtain the simple form (Equation (3)):

*k_f_* = *c*_0_·(*L*)^*a*_1_^, (3)

which, using the relation *k_f_* = 1/*t*

1/*t* = *c*_0_·(*L*)^*a*_1_^, *t* = (*c*_0_)^−1^·(*L*)^(−*a*_1_)^ (4)

can be compared with the form derived from the application of Transition State Theory (TST) [19] for the time required for a protein to overcome a free-energy barrier Δ*G^#^* at temperature *T.*

*t* = τ·exp(Δ*G^#^*/*RT*), (5)

In Equation (5), ‘exp()’ denotes ‘*e*^()^’, where is the base of the natural logarithm (~2.7183), *R* is the universal gas constant (8.314 J·mol^−1^·K^−1^), and τ is the elementary time for the inclusion of a residue into the growing secondary structure (including the time for liquid water to rearrange around the polypeptide chain), estimated at ~1–10 ns for proteins of ~100 residues, which is the average length of proteins in the sets from this study [1,26].

Through detailed physico-chemical analysis of the protein folding process, a larger group of authors [1,20,21,26,27,28,29,30] proposed functional forms for estimating the time required for protein folding. The basic forms are given by Equation (6) or (7):

*t* ~ τ_s_·*L^N^*, (6)

*t* ~ τ_s_·exp[*N*·ln(*L*)]. (7)

In Equations (6) and (7), the characteristic time constant τ_s_, associated with the time required for secondary structure rearrangement, is also included. Additional factors in the protein folding process are considered, such as the number of secondary structure elements (*N*) that are optimally positioned in the 3D structure during folding. The value of *N* is estimated to be at most *N* = *L*^2/3^/3, which, for a protein of length *L* = 100, is approximately 7.2 [1]. Another estimate gives the value as *L*/15, which, for a protein of 100 amino acids, is approximately 6.7 [28].

In the models of the form of Equation (8) (Section 3.2) developed in this study (Table 1, Table 2, Table 3 and Table 4, including Supplementary Tables S1–S4), the value (–*a*_1_) from Equations (4) and (8) ranges from 4.5 to 7.1 (Table 1, Table 2 and Table 3) for models developed on smaller datasets. For the models in Table S4 (parts C–E), developed using the larger protein sets S77, S80, and S111, these values are 4.8, 6.4, and 6.2, respectively. The values of *a*_0_ and *c*_0_ from Equations (1), (2) and (8) determine the pre-exponential factor in the calculation of *k_f_*. This contribution is estimated for the folding of one amino acid residue to be ~10^12^ s^–1^ (Figure 3 in ref. [28]), which for a protein with a length in the range ~50–150 residues would give a range of 5·10^13^–1.5·10^14^. The average value of *a*_0_ in the models in Table 1, Table 2, Table 3 and Table 4 and S4 (parts C–E) is 30.4, giving a value of c_0_ in Equation (3) of 1.5·10^13^ s^–1^, which is in good agreement with the physical theory.

Given the complexity and diversity of the proteins and datasets used to model protein folding rates in this study, as well as the experimental error in measuring folding times (rates), the agreement with the model derived from the physical theory of folding is satisfactory. A model that more closely reflects the original theoretical concepts can facilitate understanding of protein folding and misfolding processes. Improved knowledge of factors important in protein folding can advance understanding of developmental diseases [31], enhance diagnostic and therapeutic procedures in neurodegenerative disorders [32] and other diseases [33,34], support the treatment of cancer and epithelial tumour cells [35], and help reduce the inflammatory response associated with protein misfolding [36]. Furthermore, folding and misfolding are closely linked to the aggregation of macromolecules and proteins, making it important to understand the relationship between attractive electrostatic interactions and intermolecular repulsive forces [37].

### 2.7. Influence of Variation in Experimental Data on the Quality of Protein Folding Rate Models

When we analysed the four tools, we found differences in experimental folding rates and in the lengths of protein sequences used to build the protein folding rate prediction models. As there are three different training sets (two of the four methods used the same training dataset, S80 [7]), we created an overlapping dataset for each pair of these three sets and compared them in Table 6. For each pair of proteins with the same PDB codes, we compared the sequences and their lengths, as well as the experimental protein folding rates ln(*k_f_*). We considered two proteins to have non-identical values of ln(*k_f_*) if the difference was at least 0.01. For each overlapping pair of datasets, we identified the number of identical and non-identical pairs of protein sequences, as well as the range in which the difference in amino acid sequence length occurs. For proteins with identical PDB codes, detected differences in the experimental values of ln(*k_f_*) and in their primary structures are given in Tables S1–S3 (part G) and Tables S1–S3 (part H), respectively.

The data in Table 6 summarise the observed differences in sequence length and folding rate values for proteins from the S77, S80, and S111 sets. We compared pairs of proteins sharing the same PDB code across all three sets. The smallest average differences in sequence length occur between sets S80 and S111, while the number of proteins in which these differences were detected is approximately equal for each of the three pairs of protein sets. The number of proteins in which the experimental values of ln(*k_f_*) differ is much greater, ranging from 0.01 to 3.4 on a logarithmic scale. The smallest average difference in experimental values is observed between sets S77 and S80.

The reasons for these discrepancies are partly due to the chronic lack of sufficiently high-quality and reproducible data, as measurements related to protein folding are very demanding and conducted by a small number of laboratories. Consequently, folding rate measurements for some proteins were performed at slightly different temperatures [21], and when measurements were taken at several temperatures, the value obtained at the temperature closest to 25 °C was used. Additional sources of non-uniformity include measurements of folding rates at slightly different solution pH values [38]—most often at pH 7, less frequently at pH 5.5, but for several measurements this information cannot be determined from the original publications. Finally, there is variation in the composition of the solution and in the use of denaturing agents (urea and guanidinium chloride are most commonly used) [38].

Although Table 6 shows that differences in the experimental data of proteins used to develop models for predicting protein folding rates are often present, they appear to be randomly distributed across all protein sets. Therefore, they do not seem to have a crucial impact on the models developed using individual sets. It is particularly important that these differences do not significantly affect simple protein folding rate models based on protein sequence length ln(*L*), even though protein length is the only input parameter in these models. This can be attributed to the fact that both protein sequence length and experimental values of the protein folding rate are entered in these simple models on the logarithmic scale, which makes the deviation of the experimental values less pronounced than if these values were entered on a linear scale. However, it would be desirable to exclude or reduce those influences that do exist. Therefore, creating more precise and better curated datasets remains a challenge and a goal for the future.

### 2.8. Importance of Proper Selection of the Model Validation Procedure and Definition of Model Applicability Domain

The validation indicators of model quality obtained or calculated during the fitting procedure are not objective measures of the quality and stability of the model for predicting protein folding rates. Furthermore, these are not indicators derived from the cross-validation procedure, which only partially—and not entirely without bias—imitates the prediction process on independent examples (proteins). This is especially true if the model was developed and optimised using small training sets and includes many input variables that may be significantly inter-correlated.

Results from examples of multivariate methods using multiple input variables (structural descriptors) based on protein structure do not demonstrate sufficient ability to generalise to new proteins. Validation and measures of model quality obtained from one or more independent, randomly selected external sets of proteins (test sets) are significantly more stringent and objective indicators of model quality and stability, as shown in Table 1, Table 2, Table 3 and Table 4. Models based on protein sequence length appear more stable in prediction on independent, randomly selected test sets and show greater generalisation ability. These favourable properties of simple models based on protein sequence length persist despite some variation in experimental data on protein sequence and folding rate across different publications and databases.

An important characteristic of QSAR models that is considered [15] is the applicability domain. It would be useful to apply this practice to protein folding rate models as well, at least in a minimal form. In general, a minimal applicability domain can be defined with respect to the range of protein sequence lengths in the training set. This is partly implemented in several methods by defining the minimum length of the protein for which a prediction is provided, usually 30 or more often 50 amino acid residues. Additional criteria could include protein similarity, allowing analysis and recommendations regarding the suitability of the model with respect to similarity to proteins in the training set. It can be assumed that the prediction will be more accurate if the protein is more similar to one of the proteins in the training set. A more detailed elaboration of the concept of defining the applicability domain of the model is planned for future research.

### 2.9. Possibility of Improving Protein Folding Rate Models Using Predicted 3D Protein Structures

In addition to protein folding rate models based on descriptors calculated from protein sequence and predicted secondary structure, models using information from 3D structure, such as contact order distance and similar descriptors, are most promising for improving this type of prediction. Until recently, this was very difficult because such models could only be applied to proteins with experimentally determined 3D structures. However, with advances in the quality, availability, and speed of 3D structure prediction methods such as AlphaFold [39] and other modern methods [40,41,42,43,44], improvement of protein folding rate models is now likely. These algorithms have enhanced 3D structure prediction and applications [45], shifting the field from static to ensemble structure prediction [46].

Improved models for predicting protein folding rates, which can now incorporate readily available 3D structural information, could provide valuable input for encoding energy landscape and transition-state information. They may also find application in deep learning frameworks for dynamic modelling as auxiliary descriptors, physical constraints, or weak supervision signals.

## 3. Materials and Methods

### 3.1. Web Servers

The following web servers, which require only the protein sequence as input, are currently operational for predicting protein folding rates: FRTpred [8], FOLD-RATE [10], FoldRate [11], and Pred-PFR [12]. Four other servers used in an earlier comparison of protein folding rate prediction methods [9] were available online in 2014 (PRORATE [46], SFoldRate [7], SWFoldrate [47], and SeqRate [13]), but these are no longer operational. The K-Fold method [16], which is based on the 3D structure, is also in use. However, the application of K-Fold is limited and cannot provide a prediction for every protein chain for at least two reasons: (1) the 3D structure of the protein is required, and (2) many proteins with known 3D structures have one or more breaks in the structure. For such proteins, the method or server reports an error in the input data and cannot complete the output or the prediction of the folding rate.

The web server FOLD-RATE [10] uses an initial set of 49 descriptors, representing diverse average amino acid properties, to develop multivariate linear models for: (1) a complete mixed set of 77 two- and three-state proteins and (2) three subsets of proteins corresponding to three classes (all-α, all-β, and mixed). These structural classes are defined by the amount and distribution of the two regular secondary structures, α and β, within the sequence. Each of the four models is based on different subsets of molecular descriptors (i.e., average amino acid properties). FOLD-RATE models predict folding rates of 77 proteins with correlation coefficients of 0.99, 0.97, 0.90, and 0.96 for α, β, mixed class, and all proteins, respectively. The model developed using all 77 proteins (the global model) does not use structural class information [10]. The FoldRate server [11] is an ensemble predictor formed by combining three individual predictors based on properties extracted from the amino acid sequence of the protein. These three predictors were optimised on a set of 80 proteins and combined into one, yielding a correlation coefficient of 0.88 between experimental and predicted protein folding rates.

Similarly, the Pred-PFR [12] (Predicting Protein Folding Rate) server uses multiple linear regression with seven combined predictors. The FRTpred server [8] predicts the logarithmic protein folding rate value and folding type from the provided sequence for non-two-state (N2S), two-state (2S), and combined (2S + N2S) proteins. Table 7 summarises key aspects of the four sequence-based web servers, including their training set sizes, input, and output formats.

### 3.2. Datasets of Proteins and the Form of Developed Models

In this study, we examined the results from four web servers that predict protein folding rates. We used three sets of proteins with experimentally determined folding rates and known sequences, dividing them into subsets based on dataset intersections (∩) or differences (\) for further analysis.

Dataset S77 (Table 7), comprising 77 proteins, is taken from the web page and paper related to the FOLD-RATE server [10]. The second dataset, compiled by Ouyang and Liang [7], consists of 80 proteins (S80), with 45 folding with two intermediate states (TS) and 35 folding with three or more intermediate states (MS). This dataset includes 18 all-α, 32 all-β, and 30 mixed-class αβ proteins. The experimental folding rates of these proteins range from ln(*k_f_*) = −6.9 to ln(*k_f_*) = 12.9. Notably, this dataset was used by the FoldRate [11] and Pred-PFR [12] servers for model training. Finally, dataset S111, consisting of 111 proteins, is used as the training dataset for the FRTpred server [8].

Each of the three datasets we used contains proteins with known experimental folding rates and protein sequences. However, we observed that reported experimental data may differ between datasets, which is a key aspect of our analysis. To compare predictions from various protein folding servers and assess the impact of differences in experimental data across the four datasets, we began with three primary datasets: S77, S80, and S111. We then categorised proteins based on their presence or absence in different datasets according to their PDB codes. To illustrate how these subsets were created, consider two general sets, A and B. For each pair of datasets (A, B), we defined four corresponding subsets:

A ∩ B—proteins that appear in both sets A and B, using data from set A.

B ∩ A—proteins that appear in both sets A and B, using data from set B.

A\B—proteins found in set A, but absent from set B.

B\A—proteins found in set B, but absent from set A.

**Figure 2:**
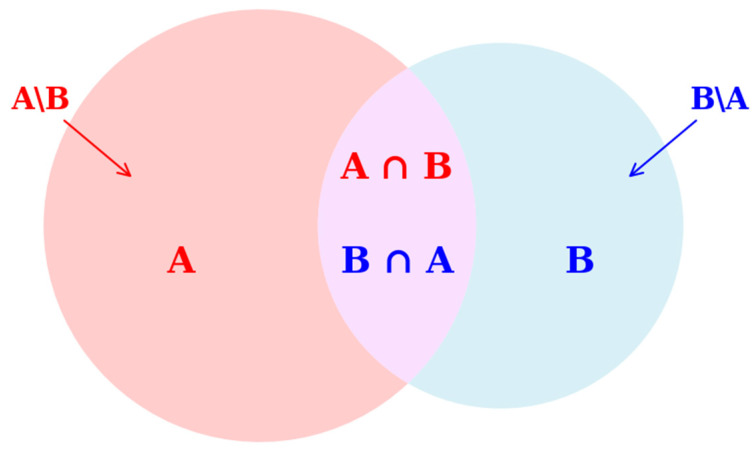
Graphical representation of subsets.

Figure 2 provides a graphical representation of this categorisation.

Combining the three sets into subsets in this way resulted in a total of 12 subsets. We then analysed the results from web servers for predicting the protein folding rates across these subsets, as well as the primary sets S77, S80, and S111, and developed our own linear models for comparison. By dividing the datasets in this manner, we obtained separate sets: the intersection (∩) subset was used as a training set for model development, while the difference (\) subset served as an external set (test set) for verifying or testing the accuracy of model predictions.

In addition, to evaluate the accuracy of the model in predicting ln(*k_f_*), we used an independent set of 30 proteins (S30) created by Manavalan and Lee [8], which was also used to validate and compare the models included in the FRTpred server. This is a new test set, in addition to the previously described test sets, which are created from pairs of sets S77, S80, and S111 as difference subsets (\).

The division of the protein sets described above was carried out to obtain independent test sets from the limited number of proteins for which experimental data on folding rates are available in the literature, in order to more rigorously validate the predictive quality of the existing and new models developed in this study. We did not initially consider the similarity between proteins in the original protein sets (S77, S80, S111, and S30), nor in the subsets we created, so as to conduct the test under conditions as similar as possible (including mutual similarities between proteins) to those prevailing in the literature.

The similarity between proteins in the data set can affect model performance. Therefore, it is important to check the level of similarity to assess whether it significantly affects the parameters by which we express model quality. We conducted the similarity analysis of these data sets using two widely used algorithms, MAFFT [48] and T-Coffee [49]. We performed the analysis for the individual sets S30, S77, S80, and S111, as well as for the inter-sections of datasets S30–S77, S30–S80, and S30–S111. The results of the analyses are presented in Supplementary Table S5 (parts A–G), which show that the percentage of protein pairs in each of the mentioned sets (and with both methods in the range) is from 0.3% to 3.2%.

Of the ten models, four are based on predictions made by web servers (MS.1–MS.4), while the remaining six are our linear models, referred to as ‘Our-Model’ followed by numbers 1–6 (acronyms MO.1–MO.6). All linear models have the form given by Equation (8):

ln(*k_f_*) = *a*_0_ + *a*_1_·ln(*L*) (8)

where ln(*k_f_*) is the natural logarithm of the experimental protein folding rate and *L* is the protein length (the total number of amino acid residues in a protein), both as given in the corresponding protein set or subset.

### 3.3. Statistical Measure of Model Quality

The accuracies of protein folding rate models are typically reported in the literature (e.g., [9]) using the correlation coefficient or Pearson’s correlation coefficient (PCC) and the root mean square error (RMSE) as measures of model quality, calculated between the experimental and estimated folding rates (i.e., folding rates estimated by the model). Sometimes, the Mean Absolute Error (MAE) is also used as an additional measure, although it provides similar information to RMSE. In this study, PCC and RMSE were calculated as statistical measures of model quality, and for some comparative analyses, MAE was also calculated. These quality measures are consistently calculated and reported in the literature alongside protein folding rate models.

To assess the quality of the model in LOO CV and external prediction (PR) procedures, we also reported the corresponding RMSE and PCC values, which are commonly used in QSAR and QSPR modelling [14]. In the field of protein folding rate modelling, LOO CV measures (here denoted by the index ‘CV’) are often reported. However, predictive quality measures denoted by the index ‘PR’ (i.e., PCC_PR_ and RMSE_PR_), calculated on an external (test) set, are very rarely used in the validation of protein folding rate models.

## 4. Conclusions

Analysis of prediction quality using four available and functional web servers for predicting folding rates ln(*k_f_*) for new proteins (i.e., those not used in the development or validation phases of each method) showed lower accuracy and reliability for all methods than reported in the original publications describing them. For the FOLD-RATE method [10], significantly higher RMSE_PR_ values were observed (see Table 1 and Table 2).

Interestingly, our simple models (labelled MO in Table 1, Table 2 and Table 3), based solely on protein sequence length, demonstrated accuracy comparable to the best methods available via web servers and analysed in this study. These models also exhibited a consistent error rate across all sets used for external validation, accounting for differences in the experimental data of the various protein sequence sets analysed. Previous literature has identified protein length as one of the most important factors influencing the folding rates of two-state folder proteins. However, we have shown that the logarithm of protein sequence length is a universal predictor of protein folding rate and, in predictions on new proteins, demonstrates accuracy comparable to or better than that of leading models in the field. Equally important, these simple models based on the logarithm of sequence length show good agreement with models derived from the basic concepts of the physical theory of protein folding, which is based on transition state theory.

To advance research in this area, it is important to establish a more precise definition of experimental data, ensure their correct use in publications, and apply much more rigorous validation of models on external sets. External validation procedures are rarely used in the development of models relating protein structure to folding rates, but are routinely applied in QSPR/QSAR modelling [15]. We therefore conclude that protein folding rate prediction models can be significantly improved by adopting the stricter model validation procedures recommended and applied in QSPR/QSAR modelling.

## Figures and Tables

**Table 4:** Statistical measures of folding rate models (evaluation metrics) from the literature [7,8,11,12] developed on the S30 dataset (30 proteins), and models from this study based on ln(*L*) developed on the S77, S80, and S111 datasets ^a^.

Model No.	Data Set	n	RMSE_PR_	PCC_PR_
MS.1g ^b^	S30	30	5.77	0.223
MS.2g	S28 ^a^	28	2.50	0.795
MS.3g	S28 ^a^	28	2.42	0.807
MS.4g	S30	30	2.22	0.858
MO.1c	S30	30	2.84	0.709
MO.2c	S30	30	2.83	0.709
MO.3c	S30	30	2.99	0.709
MO.4c	S30	30	2.98	0.709
MO.5c	S30	30	2.83	0.709
MO.6c	S30	30	2.84	0.709
MO.7 ^c^	S30	30	2.97	0.709
MO.8	S30	30	2.83	0.709
MO.9	S30	30	3.00	0.709

^a^ S30 is the set of proteins used as the independent set for the server FRTpred. The set S30\S80 contains 29 proteins because protein 1HCD was included in both sets. However, one protein with *L* < 50 amino acids was excluded, resulting in a final test set for models MS.2g and MS.3g containing *n* = 28 proteins. ^b^ Models MS.1g–MS.4g are models MS.1–MS.4 from Table 1, Table 2 and Table 3, applied to predict folding rates of the protein set S30 (or S29 or S28, as described above). ^c^ Models MO.7, MO.8 and MO.9 have the general form of Equation (1), developed on sets S75 (with one copy of each duplicated protein, 1UBQ and 2A5E, removed from S77), S80, and S111, respectively. Details of these models are provided in Table S4, parts C–E.

**Table 5:** PCC values of models from original publications for the data sets of the four prediction tools.

Tool	FOLD-RATE [10]	FoldRate [11]	Pred-PFR [12]	FRTpred [8]
PCC	0.87 ^a^ 0.99 ^b^	0.88	0.88	0.795–0.838 ^c^ 0.727–0.764 ^d^
RMSE		2.03	2.03	1.948–2.123 ^c^ 2.593–2.759 ^d^

^a^ Result with respect to proteins of unknown structural class. ^b^ Result with information about structural class/type of protein. ^c^ PCC range for several ML algorithms in the case of N2S (non-two-state) proteins. ^d^ PCC range for several ML algorithms in the case of 2S (two state) proteins.

**Table 6:** Comparison of protein lengths and experimental protein folding rates for pairs from three different training sets used in previous studies.

Data Sets in Comparison	(S77, S80)	(S77, S111)	(S80, S111)
size of the overlapping dataset	50	40	51
protein sequences			
number of identical pairs	38	29	41
number of nonidentical pairs	12	11	10
range	1–121	1–119	1–10
mean difference in length of nonidentical pairs	14.0	16.4	4.7
experimental value of ln(*k_f_*)			
number of identical pairs	17	8	10
number of nonidentical pairs	32	32	41
range	0.01–1.65	0.01–3.40	0.01–3.32
mean difference in ln(*k_f_*) of nonidentical pairs	0.20	0.68	0.68

**Table 7:** Input and output formats of functional web servers for prediction of protein folding rate.

Tool	FOLD-RATE [10]	FoldRate [11]	Pred-PFR [12]	FRTpred [8]
training set size	77 (S77)	80 (S80)	80 (S80)	111 (S111)
input format	plain sequence	plain sequence	plain sequence	plain sequence
additional input kinetic state	structural class no	no no	no no	folding type no
outputs	residue composition, structural class, folding rate in natural logarithm	folding rate in natural logarithm, half folding time	folding rate in natural logarithm	folding rate in natural logarithm, folding type, probability of folding type

## Data Availability

The original contributions presented in this study are included in the article/Supplementary Materials. Further inquiries can be directed to the corresponding authors.

## Supplementary Materials

The following supporting information can be downloaded at: https://www.mdpi.com/article/10.3390/ijms27072968/s1.

## Abbreviations

The following abbreviations are used in this article:

ERT	Extremely Randomised Tree
FOLD-RATE	Web server from [10], http://www.iitm.ac.in/bioinfo/fold-rate/ (accessed on 10 February 2026)
FoldRate	Web server from [11], http://www.csbio.sjtu.edu.cn/bioinf/FoldRate/ (accessed on 10 February 2026)
FRTpred	Web server from [8], http://thegleelab.org/FRTpred (accessed on 10 February 2026)
K-Fold	Web server from [16]
LOO CV	Leave-One-Out Cross-Validation
MAD	Mean Absolute Deviation
MAE	Mean Absolute Error
ML	Machine Learning
MO	Model-Our
MRF	Meta Random Forest
MS	Model Server
MS	Multi-State
N2S	Non-two-state
NAMs	Non-Animal testing Methods
OECD	Organisation for Economic Co-operation and Development
PCC	Pearson Correlation Coefficient
PDB	Protein Data Bank
Pred-PFR	Web server from [12], www.csbio.sjtu.edu.cn/bioinf/FoldingRate/ (accessed on 10 February 2026)
PRORATE	Web server from [46]
QSAR	Quantitative Structure–Activity Relationship
QSPR	Quantitative Structure–Property Relationship
RF	Random Forest
RMSE	Root Mean Square Error
SeqRate	Web server from [13]
SFoldRate	Web server from [7]
SVM	Support Vector Machine
SWFoldrate	Web server from [47]
TS	Two-State
2S	Two-state
